# The ‘good’ of extending fertility: ontology and moral reasoning in a biotemporal regime of reproduction

**DOI:** 10.1007/s40656-022-00496-w

**Published:** 2022-05-18

**Authors:** Nolwenn Bühler

**Affiliations:** Institute of Social Sciences, University of Lausanne, Lausanne, Switzerland

**Keywords:** Biological clock, Reproductive technologies, Biotemporality, Biomedicalization, Moral reasoning, Fertility extension

## Abstract

Since the emergence of in-vitro fertilization (IVF), a specific set of technologies has been developed to address the problem of the ‘biological clock’. The medical extension of fertility time is accompanied by promissory narratives to help women synchronize conflicting biological and social temporalities. This possibility also has a transgressive potential by blurring one of the biological landmarks – the menopause – by which reproductive lives are organized and governed. These new ways of managing, measuring and controlling reproductive time have renewed debates on the age limits of motherhood and the moral legitimacy of medical intervention into age-related fertility decline. Building on Amir’s feminist concept of biotemporality, this paper questions what happens when the ontological foundations of age-limited motherhood are disrupted by technologies which allow fertility to be extended. It discusses the reconfigurations of the ontological boundaries of the facts of life in the light of literature on reproductive technologies and temporality. Through the Swiss experience, the paper shows how medical experts are drawn into negotiating the ontological boundaries of age-limited motherhood along the binaries of the normal/pathological and the biological/social. Questioning the purpose of medical interventions in what are seen as facts of life, they produce different configurations of moral reasoning where what is natural undergoes shifts which both reinforce the normative order and subvert it.

## Introduction

The development of reproductive biotechnologies (RTs) has helped transform our conception of the ‘facts of life’ – the biological processes of human reproduction, such as “birth and procreation, the inheritance of genetic material, the developmental stages through which a child progresses” Strathern 1992b, p. 17). In Western societies, these are generally taken as the immutable natural basis underpinning socio-cultural arrangements we consider mutable. Gender and kinship are typical examples of social categories, norms and relations that are usually considered to be primarily grounded in nature, based on sex for the first one, and on blood or genes for the second (Collier & Yanagisako, 1987). However, with IVF and related technologies, reproduction has increasingly become understood as an achievement (Franklin, 1997, 2013) and the “facts of life have become more visibly partial and contingent” (Franklin, 1998, p. 106). They have “transformed from a presumed (universal, self-evident, biological and scientific) certainty into an occasion to reveal what certainty has obscured” (Franklin, 1997, p. 13). The biotechnological transformation of the basic components of the facts of life in turn affects concepts of kinship (Franklin, 2013) and genealogy (Bamford et al., 2009; Franklin, 2007) which still frame “understanding of race, personhood, ethnicity, property relations, and the relationship between human being and nonhuman species” (Bamford et al., 2009, p. 2).

Fertility decline in women is considered to be one of these facts rooted in biology. Since the 1970s, the concept of the ‘biological clock’ has described the gap between the biological temporality of fertility decline and the social temporality of family formation. Demographic studies show a trend to postpone childbearing and to have fewer children (Billari, 2008; Sobotka, 2010). Changes to family formation are located in the wider social context of women entering higher education and the workforce, as well as a general rise in divorce rates and the proliferation of family structures, such as same-sex families or recomposed families (Baldwin & Nord, 1984; Van De Kaa, 1987). The concept of biotemporality has been proposed by Amir (2007) to reflect on the gender and biopolitical implications of ‘biological clock’ discourse and practices. Through a joint reading of Foucault, Deleuze and Butler, Amir shows that ‘the biological clock’ is a new, temporal mechanism regulating reproduction. She shows how this temporal regime emerges in a historical moment when gender roles and the norms of family formation appear to be crumbling. By rooting temporal norms in the biology of women, it helps maintain a naturalized understanding of physical gender differences anchored in reproduction and reinforces a naturalized vision of the life course, in which motherhood is an inescapable stage of women’s lives. The ‘biological clock’ thereby supports the heteronormative order (Butler, 2006).

In this paper, I build on Amir’s concept of biotemporality to explore a complementary aspect of the bio-technologization of reproduction. Focusing on the potentiality (Taussig et al., 2013) of extending fertility through RTs such as egg donation and egg freezing, I want to question what happens when the biology at the core of the temporal regulation of reproductive lives becomes unstable. In other words, what happens when the nature of age-limited motherhood loses its grounding function, to quote Strathern (1992a), and is technologized? Extending fertility is discussed mostly in bioethical terms of the good and bad of whether and how far to extend motherhood (i.e. Bittner & Eichinger 2010; Goold & Savulescu, 2009; Kortman & Macklon, 2008; Lockwood, 2011; Mertes & Pennings, 2011; Smajdor, 2008, 2009). Drawing on anthropological insights, which consider that morality and ethics are always embedded socially and politically (Fassin, 2012), I give consideration to the moral norms underlying these ethical debates which are enacted and negotiated in practices in various forms of ethical boundary work (Wainwright et al., 2007). I turn to the concept of ‘moral reasoning’ (Lakoff & Collier, 2004) to understand their local specificities and shed light on the use of the ‘biological clock’ (Friese et al., 2006) in a biotemporal regime of reproduction. The question I ask is: what norms, values and reflections are in play in the ethical problematization of age-limited motherhood when what is considered as a ‘fact of life’ is technologized and remade (Franklin, 2013; Franklin & Lock, 2003)? By turning to Switzerland as a case study, I want to shed light on both the ontological implications of these debates and the complex positions medical experts take in the (re)production of temporal gendered norms.

To address this question, I shall focus on the moral reasoning of medical experts (Lakoff & Collier, 2004) when negotiating the ‘good’ of extending fertility. ‘Good’ here is intended to capture the normative logics underpinning their legitimization of a medical intervention – whether they consider it to be morally good – to extend fertility. In order to analyze the ethicization of fertility extension, I turn to the anthropological scholarship problematizing moral reason and economies through the lens of the Foucauldian analytical perspective of biopolitics (Fassin, 2009; Lakoff & Collier, 2004; Rabinow, 1992). Lakoff and Collier’s (2004) elaboration on the notion of moral reasoning is especially useful. They define regimes of living as “congeries of moral reasoning and practice that emerge in situations that present ethical problems – that is situations in which the question of how to live is at stake” (Lakoff & Collier, 2004, p. 420). Emerging when the social and biological life of individuals and collectivities is at stake, often in response to biotechnological developments, regimes of living refer to local configurations of practical, ethical, normative, technical and political elements of practice brought into specific forms of alignment which then participate in more global biopolitics. It therefore provides a relevant conceptual and analytical framework for investigating the ethical debates and moral reasoning of medical experts when they reflect on the ‘good’ of extending fertility for women in Switzerland.

Drawing on the idea that moral reasoning provides a relevant site for observing possible shifts in the regulatory mechanisms of a biotemporal regime of reproduction, I show how the potential of biotechnologically extending fertility pushes medical experts to negotiate the ontological boundaries of the age limits of motherhood and to rethink the relationships between the individual, medicine, and society. The analysis of three distinct configurations of moral reasoning will allow me to shed light on how the nature of the ‘biological clock’ shifts in different ways through their negotiations of the normal/pathological and the biological/social binaries. This case study is useful to understand the political implications of biotechnologically extending fertility. It shows how moral negotiations about the ‘good’ of fertility extension depend on the ontological status attributed to the ‘biological clock’ and to fertility decline, as well as how they situate moral responsibility on the individual, medicine, or society. I argue that understanding this connection between the moral and the ontological is useful to understand how different configurations of moral reasoning contribute ambiguously to both reinforce and subvert the regulatory power of bio-temporality in maintaining heteronormativity.

The paper is divided into two main parts. It starts with a discussion of the literature on the biomedicalization of reproduction by focusing on its implications for the understanding of ‘nature’ in a first section, and then through the lens of temporality in a second. The second part presents a Swiss case study based on anthropological research into the biomedicalization of age-related infertility, carried out between 2011 and 2016, as a contemporary empirical example of moral reasoning in which the ontological boundaries of fertility decline are central elements in the biotemporal regulation of reproductive lives.

### The biomedicalization of the ‘biological clock’

From the outset, IVF has been envisioned as a possible medical response to age-related fertility decline and the demographic concern over declining fertility rates and childbirth postponement (Leridon, 2004). The limited success of IVF in this regard has been counterbalanced by the rise in egg donation in the 1990s. This RT rests on the division of conception work between the woman who carries the child, usually the mother-to-be, the egg donor, and the sperm provider, usually the father-to-be. While this RT was initially targeted at women with ovarian malfunction, it achieved unexpected success with older women in bringing about late pregnancies and extending motherhood beyond what was deemed possible, namely after fertility declines and even beyond menopause (Antinori et al., 1993; Flamigni, 1993; Sauer et al., 1993, 1995).

Additional technologies have since been developed to address the problem of the ‘biological clock’. Advances in cryopreservation techniques led to the entry into the market in the 2010s of ‘egg freezing’ (ESHRE Task Force on Ethics and Law et al., 2012; The Practice Committees of the American Society for Reproductive Medicine and the Society for Assisted Reproductive, 2013). This vitrification biotechnology can be used to preserve the possibility of having a genetically related child by suspending biological time (Baldwin, 2019b; Martin, 2010; van de Wiel, 2015). Another set of RTs involving ooplasmic transfer were tested at the end of the 1990s in the US (Cohen et al., 1998). Although quickly prohibited due to the risky genetic implications of the procedure (FDA, 2018), it drew scientific attention to the role of mitochondria in age-related fertility processes, namely in apoptosis or programmed cell death (Morita & Tilly, 1999; Pru & Tilly, 2001; Tilly, 1996, 2003). Building on scientific work combining anti-ageing science with reproductive biology, more experimental technologies involving autologous oogonial stem cells and mitochondria have been developed (Johnson et al., 2004; Woods & Tilly, 2015), including the RT Augment for Autologous Germline Mitochondrial Energy Transfer (Woods & Tilly, 2015). Directly targeting ageing in the reproductive cells, these RTs aim at ‘rejuvenating’, ‘revitalizing’ or ‘boosting’ so-called ‘old’ or ‘unhealthy’ oocytes by injecting mitochondria into older eggs (for more information on this case, see Bühler & Herbrand, 2022; Bühler, 2022).

These technologies contribute to the biomedicalization (Clarke et al., 2010) of reproductive ageing. Biomedicalization refers to the contemporary “complex, multisited, and multidirectional” processes of medicalization” (Clarke et al., 2003, p. 161). Compared to more classical processes of medicalization (Conrad, 1992), biomedicalization is characterized by the high importance of biotechnologies, and of economic sectors and neoliberal logics which transform it “*from the inside out*” (Clarke et al., 2003, p. 162). This has profound implications for patients, as the management of risk and optimization logics are increasingly individualized, but also for the production, distribution and use of biomedical and health knowledge. The way biotechnologies contribute to a reconfiguration of the traditional boundaries between the human and the non-human in the remaking of life and death (Franklin & Lock, 2003) is a further key feature of biomedicalization. The possibility of biotechnologically extending fertility provides a good example for reflecting on the ontological, ethical and political implications of the biomedicalization of reproduction.

#### The ontological boundaries of the ‘facts of life’

Reproductive technologies is a site where biotechnological reconfigurations of ontological boundaries are particularly visible (Squier, 2004). Their close examination is important to understand how technology is at work in the “remaking of life and death” (Franklin and Lock 2001; see also Kaufman & Morgan 2005), or the “remaking of the biological” (Franklin, 2013a) in the context of the biotechnological extension of fertility. The development of contemporary biomedicine, with its molecular and genetic tools, entails a molecularization of life: the “*style of thought” of contemporary biomedicine envisages life at the molecular level, as a set of intelligible vital mechanisms among molecular entities that can be identified, isolated, manipulated, mobilized, recombined, in new practices of intervention, which are no longer constrained by the apparent normativity of a natural vital order*” (Rose, 2007: 5–6). With the development of biotechnologies, ‘life itself’ has become a material resource that can be controlled, re-engineered, and capitalized just like any other exploitable natural resource (Franklin, 2013a).

Biotechnological changes to ‘life itself’ (Rose, 2007) challenge the cluster of binaries around the biological/social, natural/artificial, normal/pathological. What pertains to one or other domain becomes increasingly blurred, thereby generating various forms of ethical boundary work where new lines of demarcation are drawn in local practical and institutional assemblages. This provides room to think of the entanglement between the norms of the biological and the social along with their implications. The normal/pathological binary has been discussed as an example of the complex relations between the norms of a vital order and the social norms at stake in the biopolitical governance of societies (Canguilhem & Foucault, 1991). Contesting an interpretation of the pathological as a deviation from a supposedly normal biological state where medical intervention is the means to reestablish that state, Canghuillem’s work (Canguilhem & Foucault, 1991) helps us to think of the entanglement between the ontological and the politics of life in practices of knowledge production (see also Jamieson 2016; and Rose 2009). Discussing Foucault in regard to Canguillem’s work, Fassin extends the discussion of the politics of life to develop a moral anthropology, examining the constitution, in given historical and geographical contexts, of norms and values, and of the split lines between good and bad, right and wrong, truth and lie (Fassin, 2005, 2006). Drawing on Angamben (Agamben, 1997), he insists on the inseparability of *zoé* (naked life, biological matter) and *bios* (the social life of individuals) and shows how the value of life (which lives are more valued socially and how life itself becomes the greatest good) is central in biomedicalization and public health. In this sense, the valuation of life at stake in ethical debates and the ontological boundaries enacted to delineate what belongs to the biological and the social or to the normal and the pathological are always deeply political, as they reveal and may reproduce social hierarchies.

Feminist STS scholarship further provides valuable analytical tools for thinking about the entanglement of the ontological and the political. Feminist theory has built on a critique of how ‘nature’ – biological, psychological, bodily, hormonal, etc. – is used to justify gender inequalities by anchoring them in presumed universal biological and physical differences (Beauvoir, 1986; Butler, 2006; Oakley, 1972). Feminist STS has also shown how the production of scientific knowledge is deeply gendered (Haraway, 1988; Harding, 2004; Martin, 1991). The extent to which biotechnological changes to ‘life itself’ either help reinforce, or indeed subvert, gender norms and categories in novel ways has been central. RTs have widened this discussion. IVF, for example, disrupts the natural foundations of kinship and gender categories whilst contributing to the “dissolution of the biological and technical […] through which biology is not only denaturalized, but ‘cultured up’” (Franklin, 2013, p. 4). It forms part of an expanding platform which brings together stem cells research, regenerative medicine and cloning techniques. From its fixed and stable ground, biology becomes technology (Franklin, 2013); it becomes a tool for re-engineering life, blurring the distinction between cause and effect, agency and passivity, being both determined by and determining human intervention (Franklin, 2013). Yet RTs are profoundly ambivalent or paradoxical (Franklin, 2013b; McKinnon 2015) and also help reproduce kinship and gender models. By imitating nature, they transform it, but also reproduce categories and relations that are the same, and yet not exactly the same. As Franklin writes, “*IVF can be described as both debiologising and rebiologising, offering a version of biology that is bespoke, artificial, controllable, personalized, and redesignable, while also providing essentially the ‘same’ route to conception, pregnancy, and parenthood as that naturally experienced by fertile couples*” (Franklin, 2013, p. 239).

The biotechnological extension of fertility has therefore a transgressive potential with regard to gender, age and kinship norms, but may, at the same time, reinforce them. In other words, it may help denaturalize these norms and categories. However, when the stable ground of nature is left open to biotechnological transformations, social normativities may also enter the ‘body’ more deeply. By enabling postmenopausal pregnancies, egg donation, for example, blurs gender, moral, epistemological, and ontological boundaries (Campbell, 2011). The frontier between what is natural and what is not is particularly breached when menopause is biotechnologically circumvented. Historically in Euro-American countries, this biological marker is used to organize and govern reproductive lives around the dichotomy fertile/infertile. It separates symbolically and materially the reproductive phase of women’s lives from the non-reproductive. Moreover, the biomedicalization of reproductive ageing itself reconfigures our understanding of fertility decline. Similar to the logics of anti-ageing medicine where, rather than illnesses and dysfunctions being understood as consequences of ageing, the biological processes of ageing itself become the target of biomedical intervention, it is the ageing process of oocytes and the figure of ‘old eggs’ (Friese et al., 2006) which take centre stage (see Bühler 2021, 2022).

Locating age in physical functions allows chronological age to be de-essentialized; it also means that cultural values and norms gain in importance and occupy the space left with something malleable and plastic: “*The natural to the extent that it still functions as a sign of ontological existence is increasingly culturalized and open to cultural experimental forces*” (Katz & Marshall, 2004, p. 54). Whilst claiming to liberate us from biology, biotechnologisation nevertheless pushes “*our cultural values right back onto our body’s functions, hormones, brains, and faces*” (Katz & Gish, 2015, p. 56). Replacing classical binaries of normal and pathological, the focus on biological processes of ageing themselves indicates a cultural drive towards “optimization” (Mykytyn, 2008), that is an individualization of the moral responsibility of citizens to secure the “best possible futures” through the use of biotechnologies and the consumption of biomedical products (Adams et al., 2009; Rose, 2007).

Biotechnological transformations of ‘life itself’ have therefore implications for the constitution of the subject and the ways people navigate their health and reproductive lives. Feminist literature has extensively theorized about the impact of medicalization on women’s bodies and subjectivities in terms of power relations and naturalization of gender norms (i.e. Clarke et al., 2010; Martin, 2001). It is therefore important to look upstream at how medical experts negotiate and enact the ‘good’ of extending fertility; this reveals how they think of the relationships between the individual, medicine and society along with their political implications. In analyzing how fertility extension technologies contribute to change understanding of ‘old eggs’, (Bühler, 2021, 2022) shows how multiple version of the biological which can be more or less fixed / plastic are produced. However, how these transformations at the biological level relate to moral reasoning and, at the same time, possibly reinforce gender norms and inequalities, remains to be explored. Literature on temporality in biomedicine, to which I now turn, contributes to an understanding of how specific social norms relating to time regulate reproduction. Indeed, the biotechnologization of reproductive ageing and the extension of fertility relate directly to the ‘biological clock’ set of discourses and practices at work in governing reproductive lives where biological and social temporalities play a crucial role.

### The temporalities of reproduction after progress^1^

The relationship between temporality and biomedical technologies is of growing interest for the social studies of biomedicine, biotechnologies and health, due to their implications for ‘life itself’ (Rose, 2007), subjectivities, medical practice, bioeconomy and society. Biotechnologies challenge our understanding of time, for example, when cloning technology is used for reproductive purposes and disrupts the linearity of genealogy and categories of species (Franklin, 2007; Friese, 2013); when cells get a life of their own and their ageing and death are reconfigured (Landecker, 2003, 2007); when time is suspended and life made ‘latent’ as in freezing technology (Radin, 2013). Temporality is also at work in shaping individual and collective futures through biotechnological developments; much of the scientific work in this field is performed in the name of ‘progress’ or the future promises of having a child and improving or extending life. Following Brown, one can say that “technological change is therefore a process of constant oscillation between present and future tenses, between present and future solutions” (Brown, 2003, p. 6).

In their problematization of time in medicine, Jain and Kaufman (2011) show that we live in a time ‘after progress’ where futures are constantly returned to the present through imaginings, hopes and promises. They insist that the way the future is imagined and lived in medical spaces has an impact on the present, determining who should have a future and what kind of future that might be. Focusing on the ageing society, Kaufman & Fjord (2011) define progress as the “*long-held enlightenment idea that rationality and its tools can unequivocally improve life and reduce suffering*” (Kaufman & Fjord, 2011, p. 213). In contrast, the notion of ‘after progress’ “*critically reflects on the priority and effects of even more technology use in an aging society”* and on the new hierarchies, economies of responsibility and moral imperatives this may bring with it as “*technical ability and success become ethical necessity*” (Kaufman & Fjord, 2011, p. 213).

Biotechnological transformations affect an individual’s relationship to time as they have to situate themselves and orient their choices towards new temporal measures and standards emerging from biotechnological developments, for example, when the category of functional age and the ideal of healthy ageing replaces the normal/pathology binary in a way which increases individual responsibility, self-management and optimization logics (Cardona, 2008; Katz & Marshall, 2004) or when women are expected to anticipate their fertility decline (Baldwin, 2019b; Brown & Patrick, 2018; Carroll & Kroløkke, 2018; van de Wiel, 2015; Martin, 2010). The growing importance of the prenatal period in the context of epigenetics provides another example of how shifts in the scientific understanding of the genetic ‘make up’ of an individual is embedded in gender norms, when women are made responsible for the future health of their child before even getting pregnant (Warin et al., 2012). Furthermore, these biomedical developments contribute to a reconfiguration of the life course itself by transforming some of the temporal markers and boundaries used to organize and structure it (Franklin & Lock, 2003; Kaufman & Morgan, 2005; Moreira & Palladino, 2008). Examples are when postmortem or postmenopausal conception are made possible by sperm cryopreservation (Kroløkke & Adrian, 2013) and fertility extension technologies (Wiel, 2020); when the notion of brain death (Kaufman & Morgan, 2005; Lock, 2001) or the category of prognosis (Jain, 2007) blur the boundaries between life and death; and when technologies promoting longevity change the timing of death into a calculation between life time with regard to technological possibilities and age (Kaufman & Fjord, 2011).

Technologies extending fertility (Baldwin, 2019a) offer promissory discourses and empowering narratives, as assisting women in synchronizing conflicting temporalities so they can achieve motherhood at the ‘right’ time and be freed from the constraints of biology (Waldby, 2014; Martin, 2017). The promise of liberating women from their biology and associated time constraints towards a kind of ageless fertility goes hand in hand, however, with the reinforcement of norms and expectations relating to the ‘good timing’ of motherhood (see Bühler, in press). Embedded in anticipation politics (Adams et al., 2009), the ‘biological clock’ discourse is associated with a sense of urgency justified by specific female reproductive functions; it demands action in the present, and the moral responsibility rests on women’s shoulders. In her analysis of the ‘biological clock’ discourse, Amir shows how it turns the demographic concern about fertility decline into a female biological problem and an individual responsibility of women (Amir, 2007). Coining the concept of biotemporality, the author shows how the ‘biological clock’ discourse contributes to the biologisation of a temporal normative order which is gendered. As a result, the age of motherhood, which is put it at the center of public and medical attention, is individualized and biologized.

To further the analysis of this biotemporal regime of reproduction by shedding light on the importance of moral economies in regulating the normative/subversive potential of RTs, I focus on the ‘moral reasoning’ (Lakoff & Collier, 2004) of medical experts when discussing the ‘good’ of extending fertility medically in Switzerland. Medical experts work in a highly biotechnologized environment, but they also work within the framework of the Swiss regulations, modelled on the category of ‘nature’ with the underlying principle that only biotechnologies that enable what is possible without biotechnological assistance – or in broader terms ‘culture’ – should be authorized in the realm of reproduction. The biomedicalization of reproductive ageing and the development of fertility extension technologies bring issues related to the biomedicalization of ageing earlier in women’s lives than the menopause. They turn the management of reproductive temporalities into a women’s responsibility, but also reconfigure the very notion of reproductive ageing itself. Ethical discussions about the ‘good’ of extending fertility provide a relevant site to observe how the potentiality of biotechnologically transforming the natural ontology of age-limited motherhood requires medical experts to negotiate what is natural/social and normal/pathological in different configurations of moral reasoning where moral responsibility lies alternatively on individual, medicine, and society. If, following Amir, the ‘biological clock’ concept which articulates the biological and social temporalities of reproduction is a regulatory mechanism at work in reinstating a heteronormative order, looking at how medical experts actually differentiate what is social/biological and normal/pathological about the biomedical extension of fertility is an important step in understanding the political implications of these moral economies of responsibility. By approaching the philosophical questions presented above empirically, my objective is to understand how what is viewed as natural and normal by these experts not only varies but also relates to their imaginaries of desirable futures of reproductive medicine and of the place of women in society. It provides thus an interesting case to think further about the gender normative implication of the ‘culturalization’ of the biological.

## Extending fertility in Switzerland

The following case study is based on anthropological research on reproductive biomedicine and age-related infertility in Switzerland in the context of two projects funded by the Swiss National Science Foundation between 2011 and 2016: 1) Fertility and Family in Switzerland. Local Processes of Reproduction and Kinship in Transnational Contexts of Biomedical Technologies, n°10001A_130344, UZH; 2) Reproductive technologies, biological clock discourses and the extension of fertility time: gender, kinship and biopolitics of reproductive ageing in Switzerland, n°P1ZHP1_148681, UZH and UC Berkeley). The research project comprised three components focusing respectively on: (1) the production of scientific knowledge on fertility decline and the role ARTs play in it; (2) clinical encounters and experiences of involuntary childless women and couples undergoing or having undergone reproductive treatment in order to have children and build their family; (3) the legal framework and the ethical debates surrounding the possible authorization of egg donation and the introduction of egg freezing, both technologies opening up the prospect of medically intervening in the ‘biological clock’. Over the course of the project, the following data were collected: semi-directed qualitative interviews with clinicians and other experts involved in reproductive medicine (biologists and gynecologists specialized in fertility treatment (n = 10); counselors and psychologists (n = 4); ethical and legal experts (n = 2); other stakeholders (n = 5)) and with women and couples encountering fertility problems and turning to RTs (n = 34); ethnographic observations during interviews, conferences, information sessions, and at a reproductive medicine unit; and a corpus of scientific and medical articles, as well as legal and media texts relevant to the Swiss context. This paper limits itself to an analysis of interviews with medical experts^2^. However, the other data enabling a more comprehensive understanding of the debates at stake also inform the analysis. Ethical approval from the ethical commission of the Vaud University Hospital (now CER-VD) was obtained on Sept. 21, 2011. All identifying information was removed or substituted in order to provide the informants with anonymity^3^.

Questions about the medical, ethical and social implications of fertility extension technologies for medical practices and the legal framework regulating them took on greater significance in Switzerland in the 2010s. This renewed interest was triggered by the possibility of some RTs – egg donation and freezing – to be legally authorized and work as medical assistance to extend fertility. The empirical importance of ethical debates on the use of RTs to extend fertility and the age of motherhood is a reason I decided to focus my analysis on the moral reasoning of medical experts in order to shed light on the biotemporal regime regulating reproduction in Switzerland. By European comparison, Switzerland is usually considered a rather conservative country when it comes to reproductive biomedicine, which is strictly regulated. It is often compared to Germany and Austria, two neighboring countries which also have somewhat conservative legal frameworks, in contrast with Spain or Belgium which promote individual rights to reproductive medicine, rather than the protection of the family and the well-being of the child, as in Switzerland (Engeli, 2009; Pennings, 2002). The Reproductive Medicine Act (RMA) was passed in 2001 following a long decade of fierce debates about which procedures should be allowed or prohibited and more generally about the role of RTs in the making of families. A climate of fear and mistrust towards possibilities opened up by new biotechnologies surrounded the implementation of the regulation (Engeli 2010; Schmid 2009). Twice initiatives against RTs were launched by conservative and religious parties. They sought to ban any extra-corporeal conception in the name of the protection of human dignity, the family and the well-being of the child (Engeli 2010). The RMA, adopted by popular vote and passed in 2001, reflects the compromises found by the different parties involved in its elaboration. It restricts the use of RTs to medically diagnosed infertility and is based on a naturalistic framework, with the underlying idea that RTs should not make possible what is not possible without medical technology. In other words, they should mimic nature but not substitute it.

As a result, egg donation, one of the most widespread technologies used to extend fertility, is prohibited, unlike its male counterpart, sperm donation, which is authorized for married couples, based on the Roman principle that motherhood is certain, unlike fatherhood, considered to be always uncertain (Manaï, 2008). The possibility of authorizing egg donation in the context of a legal revision, along with the entry into the market of egg freezing in 2013, a technical possibility not envisioned when drafting the law, has renewed debates on fertility limits and the legitimacy of a medical intervention for reasons of reproductive age. What was especially at stake in the medical debates was the legitimacy of a medical intervention capable of circumventing age-related infertility. This raised the central question of the ontological status of the kind of infertility associated with age. Is age-related infertility a social or a medical problem? Is it a medical condition justifying the use of assisted reproductive technology or a physiological process, a life stage that women have just to accept and reproductive biomedicine stay clear of? What would legitimize medical intervention to extend fertility?

Both technologies can be seen as fertility extension technologies which disrupt the ontology of age-limited motherhood. In the RMA, there is no definite and explicit limits mentioned; nonetheless it states that “*ARTs may be used in couples who, on the basis of their age and personal circumstances, are likely to be able to care for and bring up the child until it reaches the age of majority*” (RMA, article 3.b). Formulated in a gender-neutral way based on the undifferentiated unit of the couple, the statement rests on a logic of intergenerational relations, where parents take care financially, physically, emotionally, of their progeny at least until the age of majority (18 in Switzerland). However, the explanatory document accompanying the vote specified that since egg donation is prohibited, there is no need to establish explicit age limits for motherhood, as they are fixed by biology itself (Delamuraz and Couchepin 1996: 245). As a consequence, if the intergenerational logic is valid in determining the age limits of fatherhood, the biology of fertility decline is used to determine the age limits of motherhood. The social norms determining the temporal limits of the life stage of motherhood are anchored in female biology and thus naturalized, while fatherhood is instead determined legally through an implicit intergenerational social contract with the state in regard to their role as breadwinners. This differential reinforces the gender representations of fathers as financial providers responsible for families, while the woman’s role is to reproduce the family in the biological sense.

However, the biological temporality proper to reproductive ageing has no clear-cut limits. It is by definition a process unfolding over time. Fertility potential is known to decline more sharply after 35 and ‘eggs’ age long before menopause. If statistics establish some thresholds at a populational level, they may also vary greatly among women at the individual level. It is also deeply relational, as successful fertilization results from the combination of multiple parameters, starting with the fertility potential of the partner. In the Swiss context, where access to RTs is restricted to pathological cases, the prospect of intervening technologically on it challenges the legitimacy of a medical intervention. As a result, age limits have become a matter of concern for practitioners. This pushes them to think about the ontological status of age-related fertility decline. As pathology defines access to RTs, the question as to when infertility is considered pathological or becomes normal emerges as crucial. As the following analysis of three distinct positionings about the ‘good’ of extending fertility will show, the normal/pathological status of fertility decline is central in the moral reasoning of medical experts. This central binary is also set against another binary: biological/social, through the category of nature which refers ambiguously sometimes to what is normal socially and sometimes to what is biological. The three positionings presented below illustrate key features of the three “congeries or moral reasoning and practice” that I identified in the field. The three positionings are emblematic of three ways of considering the relations between the individual, medicine and society depending on their understanding of the ontology of age-related infertility; it shows how differently they place the weight of moral responsibility on the individual, medicine and society, depending on their vision of the ontology of the biological clock.

### Between normal and pathological

Dr A is a renowned authority in the field of reproductive biomedicine in Switzerland and a practitioner established for a long time in a private practice in a Swiss city. I met him in his comfortable and elegant medical office centrally accommodated in an old building in a large Swiss city. I will draw on my detailed discussion with him to illustrate the first form of moral reasoning: this rests on a specific understanding of age limits based on a biological temporal order used to determine what is normal and what is pathological and therefore legitimizes (or not) a medical intervention to extend fertility. This medical expert explained to me how the decision to intervene with RTs should be taken by drawing on several exemplary fictional ‘borderline cases’ as he calls them. These cases highlight interestingly the room for maneuver of doctors and the moral values and logics underlying their clinical practices when they need to determine the legitimacy of medically extending fertility:

*I take a sensitive example, which is the daily bread of the clinician. It is a couple, she is 44 years old and he is, let’s say 28 for example, but he has semen of disastrous quality. Because of his sperm problem the couple should undergo IVF. Yet the pregnancy rates in IVF with the oocytes of this woman fall to zero from age 43. There is no pregnancy brought to term. Ok there are exceptions, but anecdotally, and in principle, many centers set a limit at 43 and I, personally, don’t do any IVF for somebody who’s older than 43, because it just does not work, so I won’t draw people down a path where they will spend energy, hope, and money for something that won’t work. I think it’s unfair. But the problem is that this woman cannot have any IVF because she is too old and he cannot have any children because his sperm is too bad. It is typically a couple, perhaps if she had a husband who had sperm of wonderful quality, she would have children, and if he had a younger wife, then he could undergo IVF and they would have children. So, from time to time there are unfortunate relationships. So, should egg donation be considered in cases like this one? I would say no, because… it is a subtle reasoning, he has the right to IVF, but she cannot because she is too old. It is not because she is ill, it is a borderline situation, a limit situation.* (Dr. A. 05.12.2011)

Here the practitioner evaluates the legitimacy of turning to egg donation based on the statistical understanding of what is normal and pathological. As statistics show that after 43 success of IVF without egg donation is minimal, a medical intervention would not be legitimate. According to this logic, it is not the suffering, desire or intent of the couple, nor his medical condition that prevails – for example, that his sperm is of poor quality – but the fact that age-related fertility after 43 cannot be regarded as an illness necessitating a medical intervention. This case illustrates how the restriction of access to RTs based on a medical or pathological condition, which is at the core of the legal regulation, is mobilized to assess the ‘good’ of a medical intervention. Age-related fertility decline after age 43 is not considered to be an illness based on the declining curve of IVF success rates. It reflects rather what is considered as the natural path of fertility potential. Without a fertility extension technology – egg donation – this woman would not get pregnant. This creates a distinction between two kinds of infertility: one which is pathological and for which a medical intervention is legitimate; one which is normal, after 43, and for which egg donation should not be used. The reflections of this doctor are also based on a gendered understanding of age limits as inherently biologically limited for women. It is important to note the asymmetry in the couple as age would not be a problem if their situation was reversed and the man was older. In other words, the normal/pathology binary serves also to reinforce gender and age norms in couples where women are younger than men.

To illustrate his position more clearly, the doctor turns to the example of anti-ageing medicine to explain how he sees the role of reproductive medicine in age-related infertility:

*Dr. A.: If they* [the older women turning to egg donation abroad] *find people who agree to do it, fine, I cannot do anything to prevent this, but it is not my vision of medicine. There are analogies, for example the aesthetic surgery of aging, I am not in favor of that. I am not speaking about aesthetic surgery in general, because sometimes, there are really important problems for the persons. Again, the boundary is critical. Typically, when the aesthetic surgery means removing wrinkles, this kind of thing, I am not in favor, it is only my personal opinion, but I think that medicine has nothing to do with this.*

*Researcher: Is it as though you were going beyond some natural limits?*

*Dr. A: Yes, yes, but no. Sometimes we go beyond a natural limit. That means, an appendicitis, it kills the patient quite often, so we intervene surgically and I think that we are right to do it. A bacterial infection, we give antibiotics, thus we do not let nature takes its course.*

*Researcher: In fact, even in reproductive medicine in general this is the case.*

*Dr. A: Exactly, but we can situate, we can define what a disease is. In my opinion, this is the common denominator. And disease is what deviates from what is happening to everybody. In fact, a fifty-year-old woman, most fifty-year-old women do not have children, therefore medicine should not substitute Nature in this sense. (Dr. A. 05.12.2011)*

The comparison Dr. A. makes between fertility extension technologies and anti-ageing aesthetic surgery draws attention to a similar logic at work in both cases. The comparison with removing wrinkles reduces fertility extension to an unnecessary and superficial intervention, or a luxury procedure, in opposition to ‘real’ medical problems targeting a pathological condition. More than the crossing of a natural boundary in itself, which is considered perfectly legitimate in many cases when it is considered pathological, what seems problematic for this doctor is that age-related infertility is a normal or physiological process. This logic becomes even clearer when Dr. A. specifies how he understands the role of reproductive medicine:

*I became a doctor in order to cure sick people, to cure disease, not to put myself at the service of social evolution, which now brings us to having children later. This is none of my concern. I mean that medicine is not concerned with this. It concerns the way we choose to allocate the different parts of our lives. […] I guess that if a woman about 45, 46, 48 doesn’t get pregnant, then it is because of her age. Medicine does not have anything to do with this. […] If a minority cannot do what the majority of women can, it is not fair, so medicine should intervene. […] But it is possible to define what is pathology, this should be the common denominator. Then pathology is what does not affect all the people. Most 50-year-old women do not have children, so medicine should not substitute itself for nature. (Dr. A. 05.12.2011)*

Here, we can clearly see a boundary being established between an age at which most women are infertile, where reproductive medicine should not intervene, and an age where its intervention is legitimate in order to re-establish the normality of a life course. The boundary between what is normal and pathological is based on a statistical understanding of the biology of reproduction, as what the majority of women’s bodies can and can’t do at a certain age. RTs are seen as tools enabling the re-establishment of the norm of young, fertile bodies, but not to help older women. Those are seen as having to take responsibility for the consequences of what is considered their individual choices, choices situated in the context of ‘social evolution’ which are considered beyond the scope of reproductive medicine. In this sense, the strong association of youth and fertility, ageing and infertility, is strengthened and naturalized through the normativity of the biology of reproductive ageing itself. In addition, extending fertility is seen as going against nature in the sense of biological processes. The role of reproductive medicine is thus strictly consigned to support a normal life course based for women on the biological phases of reproduction; the life stage of motherhood being determined by biological temporality and not by deeper structural socio-demographic changes.

### Adjusting biological and social temporalities

The second position is less common among the experts I interviewed but no less interesting and complex. I turn to my discussion with two women obstetrician-gynecologists, Dr. B. and Dr. F., working in the private sector. Like Dr. A they are well-known in the field and have extensive professional experience. They both have had families and demanding careers themselves, alluded to in the interviews in a sympathetic identification with the women having to face fertility decline. In their practice, Dr. B and Dr. F. deal with a mixed population of patients with fertility problems and a general population of gynecology. Dr. F. started our discussion by explaining how social changes impact the timing of family formation. The postponement of childbirth is linked to an increased risk of age-related infertility:

*One needs a partner, to be in couple, to complete a family project. Fertility rates are higher before 30 but around twenty nobody’s ready. It is the price of our emancipation, and the price too is that men are very anxious about having a child, men are brakes, they are not ready before 40 or even later. (Dr. F. 02.05.2012)*

In this quotation, she highlights how two temporalities conflict. Biographical temporality is dictated by the social norms of the heterosexual couple and inscribed in broader social transformations brought about by female emancipation. It is seen as out of synch with the biological which dictates that the best moment to have a child would be in the twenties. This echoes widespread discourses on the ‘biological clock’ which suggest that postponing starting a family because of higher education and entering the workplace conflicts with women’s most fertile years making them too old, reproductively speaking, when they do decide to have a child, and increasing infertility. By implicitly including herself as a feminist by using ‘we’, this practitioner sees the ‘biological clock’ discourse as part of the political battle of – white, middle-class and heterosexual – women to achieve equality with men. In this sense, the postponement of childbirth is seen positively, even though it has an unwanted side effect – ‘the price to pay’ – a higher risk of infertility from reproductive ageing. The biological clock discourse also has a part in gender relations where the reproductive calendars of men and women conflict (Bessin and Levilain 2012).

Similarly, Dr. B. also sees the problem of the biological clock as a conflict between genetically-determined, limited fertility and social changes that have led women to postpone motherhood. As with Dr. F., changes that have led to an increased participation of women in studies and work are seen positively. Their reflections on the ontology of fertility decline allow them to develop their own positions on the legitimacy of intervening medically to mitigate the effects of age on fertility. These two gynaecologists observe that there is a gap between biological and social temporalities, without rejecting these social transformations as beyond the scope of reproductive medicine, as in the first positioning (Dr. A.). Rather, they both assert that reproductive medicine has to adjust to these societal transformations. Facing age-related infertile women in their practice every day and being particularly aware that IVF is powerless to help them, both practitioners acknowledge women’s distress and suffering. They especially stress women’s lack of agency at the individual level, given they have limited scope for acting personally on the multiple structural factors determining the ‘right’ time to have a child:

*It is not that they do not want a child, but it is because they are in a personal situation that does not enable them to realize this project. It means that they are not in a stable relationship, or that they had a relatively long-term partner, but they split up. And it is really tricky to tell them to think about their biological clock [the biology of fertility decline] while obviously they do not have the necessary conditions to realize their motherhood or parenthood project. It is thus really difficult at that level. (Dr. B. 25.01.2012)*

While ideally Dr B. thinks that more information on age-related fertility decline should be publicised, she acknowledges that in practice it would just stress women more. According to them, it is not that they do not want children or are delaying childbirth without thinking about it or through ignorance. It is rather that they are not well positioned socially to have children. She thus relativizes the idea of individual choice espoused in the first positioning (Dr.A.). By considering social expectations and the personal desire to have a child, she sees that the social and medical cannot be disentangled. The social ‘requirements’ determining the right time to have a child – stable couple first, work second – are an integral part of her clinical decision not to stress patients with a precautionary message that they could not heed anyway. She does not separate them, nor does she think that as a practitioner she should fight against them, she defends rather the need to adjust reproductive medicine practices to the current social norms determining reproductive timing. The social character of the stages of the life course, especially having a family, are hereby integrated in her clinical judgments.

The role of ARTs, especially egg donation, in extending fertility is also discussed by Dr. B.:

*Let’s say, I think that egg donation has happy days ahead of it, in the sense that by that time we have solved the problem of society’s evolution and women will have realized the problem, and then we will have reversed the current trend to postpone having a family, and the next generations will have children earlier and go on with their careers perhaps after a break, but have their children earlier and study later* [which would imply no need for egg donation or freezing]. *I think that we are very far from that and therefore there will be an increased demand for egg donation* [implying, until the problem of society is solved]. *And as such to shut the door to these women completely* [refusing the use of fertility extension RTs], *I think that it is just not to live with its own time, and not to be conscious of what it means. (Dr. B. 25.01.2012)*

Without defending egg donation as a perfect ‘technological fix’ (Almeling, Radin and Richardson 2014) or promoting an ideal ever-optimizable and ageless fertility, Dr. B. judges a reversal in social changes in family formation and a transformation in gender relations between men and women to be utopian goals. It might happen one day in a distant future; but it might also not. Society is seen as open to change, the possibility of reversing the current trend to postpone childbirth is imagined but remains beyond the scope of individual action. As the ability to dictate social trends seems illusory and, implicitly, not the goal of reproductive medicine, medical practitioners’ role is to help women adjust biological and biographical temporalities. This legitimation of medical intervention is not envisioned as a technophile positioning with the objective of encouraging social transformation, but because reproductive medicine is part of the world in which one lives and to which one has to adapt.

Like Dr. B., Dr. F. also thinks that little can be done to change the trend to postponing motherhood. She stresses all the structural difficulties – lack of day-care centres, brief maternity leave, lack of male support – which make it hard for women to reconcile work and family in Switzerland. But she goes even further and develops an alternative economy of responsibility. On the upper age limit for intervention she says:

*Sometimes, women need to be* [hormonally] *stimulated. I don’t close the door, because they will continue to consult anyway, because they need it. At a pinch it would be inhuman. I cannot judge. Even if we tell them that pregnancy rates are less than 5%, if we don’t try, they will have the feeling that we are shutting the door. (Dr. F. 02.05.2012)*

In this quotation the focus is on the sufferings of the involuntary childless and the disruption of the life course it creates. If, in the position Dr. A. first presented, pathology as a physical and normative dysfunction legitimizes medical intervention, here it is the human suffering generated by the difficulties of becoming a mother that legitimizes it. Independent of the reasons that led to the condition, ARTs are seen as a legitimate tool for alleviating the suffering of childless women in their forties and supporting their desire to become mothers. According to this logic, reproductive medicine is seen as potentially helping to bridge the gap between conflicting temporalities and working as a biographical catch-up tool. Instead of turning to the statistics of success rates, Dr. F. reflects on the relational level of care, i.e., the moral obligation to intervene to give hope and to accompany women on their path to motherhood (or non-motherhood). What justifies medical intervention is not the presumed pathological status of infertility but the suffering it entails – as well as a certain gendered vision of a normal female life course in which motherhood remains essential for many women. Once a technology can be used, it is unfair not to use it in order to alleviate suffering. She says:

*Clearly, techniques open the door to situations where we just had to grieve before. But all societies had their transformations. The Romans had adoption and did a lot to remedy infertility problems. I think that our values are on the move. 20 years ago, egg donation initially shocked us, but today […] Of course, what we do is not natural, it has effects, but it is really good that ARTs exist, I mean there are so many children who are well and who are really a joy for their parents, for everybody. It is a great possibility. Before, it was really hard when one lived with infertility, it was very, very difficult. (Dr. F. 02.05.2012)*

In this quotation, the human will to act technically to alleviate the pain generated by infertility is naturalized and normalized as part of human history. If this logic is part of a ‘tyranny of potential’ (Kaufman, 2013), the use of RTs to possibly enable women to extend fertility is here normalized as part of the human endeavour to alleviate the suffering of not achieving motherhood. Therefore, an ambivalent use of the category of nature can be observed. On the one hand, Dr. F. recognizes that using ARTs is ‘not natural’ as it goes against a state one previously just had to accept at a time without technological fixes. On the other hand, she naturalizes the will to act on the suffering generated by infertility as something that has always existed as part of humankind. In this sense, the distinction between normal and pathological used in the first positioning becomes irrelevant. Moreover, social transformations here are fully integrated into moral reasoning about the legitimacy of medical intervention and not opposed to them as in the first position.

However, this logic has its own limits; Dr. F. thinks that ARTs should not be used after 50, the symbolic frontier of the menopause. She justifies this limit with recourse to the idea of natural fertility and the legal obligation to care for a child until it reaches adulthood, in a similar way to how the age limits of fatherhood are legally considered. She also justifies the setting of a limit by the idea that when reproductive medicine as a third party intervenes in reproduction, it engages her own moral responsibility, as a gynaecologist, towards the ‘good’ of a potential child. In addition, she mentions the health risks to older women with pregnancy after fifty. PM pregnancies are thus imagined as particularly problematic for women and potential children. These two gynaecologists do not defend the use of ARTs as fertility extension interventions animated by the ideal of ageless fertility or the desynchronization of the biological and social age of motherhood. They rather stress the need to set some limits to obviate being seen as a ‘sorcerer’s apprentice’, very present in Switzerland. However, they take societal transformations into account and see the moral responsibility of reproductive medicine practitioners as assisting women to adjust biological and social temporalities. By shifting the focus on pathology to the sufferings entailed by childlessness and biographical rupture, independent from its causes, and even more by inscribing the will to develop sociotechnical responses to it in the nature of humankind, they blur the distinction between the normal and pathological or the social and medical. In this sense, the role of reproductive technologies to extend fertility is legitimized at least until the frontier of the menopause at fifty.

### An illusory medical solution and a societal problem

The third position presents an additional way of relating the ontology of fertility decline to the legitimacy of medical intervention to extend fertility. It can be found in an article published in a Swiss medical journal by another clinician I also met for an interview. A renowned expert in the field, this practitioner is a member of several professional societies specializing in fertility preservation and menopause and sits on a number of ethical commissions. The article discusses the recent technological possibility of freezing eggs and of preventing age-related infertility by preserving gametes in the state, or at the age, that they were retrieved (Wunder, 2013). The author writes:

*Unfortunately, social freezing is in general not a solution for the underlying societal problems to fit in with professionally active women and having children. It only delays the existing problems. Furthermore, it creates a lot of potential new problems. A great deal more should be undertaken to offer real solutions to the underlying societal problems which are in part: pre-school education, care in the event of childhood illness, and the many weeks of school holidays, acceptance of professionally active women having children, and more job offers with a workload < 100%. Furthermore, society should be informed about the decreasing chances of pregnancy with increasing maternal (and paternal) age as well as the increasing risks of miscarriage and obstetric/neonatal complications.* (Wunder, 2013, p. w13746)

This quotation shows how age-related infertility and medically-assisted extension of fertility time are generally seen as ‘societal problems’. Switzerland is characterized later in the article by its ‘rigid structures’ and its ‘paternalistic view of the role of the woman in society’(Wunder, 2013, p. w13746). In consequence, rather than multiplying reproductive technologies to possibly extend fertility, this author makes clear that the ‘real’ problem is social, and that a ‘technological fix’ will only delay and bring even more problems. What are they? The article focuses on the “neonatal and maternal risks of primiparity at an advanced maternal age > = 40 years” and lists all the health problems related to age, but also more generally on the use of IVF-ICSI and the lack of information about the outcome of children born after oocyte vitrification. It then develops a second aspect of the question, which is ethical, by weighing the pros and cons of the possibility of freezing eggs and balancing the wellbeing of the child against the reproductive autonomy of women. The insistence on medical risks shows how the medical framing of late pregnancies is used to prevent the use of RTs to delay childbearing. It shows how the application of RTs as fertility extension technologies is undesirable, as they might bring more side-effects, risks and medical problems rather than solving the ‘biological clock’. In contrast to the second position (Dr. B. and Dr. F.) which legitimizes medical intervention by the suffering caused by a disruption in the life course and integrates societal transformations into medical reasoning, this third positioning sees medically-assisted extension of fertility time as having more negatives than positives.

In this sense, the author draws a line between the social and medical etiology of age-related infertility, as in the two other configurations of moral reasoning. A shared understanding of the social causes of age-related infertility is present in the second and third positions. However, compared to the second one, which defended the legitimacy of medically assisting fertility extension, in the third positioning society becomes targeted as the relevant site of intervention. If age-related infertility is the result of a patriarchal society and of the sexual division of labor preventing women from pursuing a career, then these problems should be the target and not fertility decline itself. This puts society outside the scope of reproductive medicine. Paradoxically, it adopts a non-medicalized view of age-related fertility decline, where the question of knowing whether reproductive ageing is normal or pathological is no longer relevant, by turning the societal causes of age-related infertility into the main problem.

The illusory character of RTs extending fertility – egg donation and freezing – attracts particular attention. They are not seen as capable of providing a catch-up on the life course as in Dr B.’s and Dr. F.’s accounts. In contrast, the false promise of technologically synchronizing biological and biographical times is seen as generating more risks than benefits. In addition, and included in the ethical aspect, there is a concern expressed about disruption to intergenerational relations which marks the emergence of ‘social risks’ in medical experts’ discourses (this notion is also found in the expert debates about the future of reproductive medicine Bleichenbacher et al., 2010). One can read:

*It has also to be considered that grandparents, who nowadays help a lot in looking after their grandchildren during the school holidays or in the case of illness, would be, in the case of delayed motherhood (after social freezing), either in a state of health incompatible with the energy of the children, or deceased. It has also to be mentioned that later on, these old parents, will for the same reasons not be able to look after their grandchildren. And we must also not forget that the resulting children could be very ashamed to have parents who could be perceived as their grandparents, possibly leading to psychological problems. (*Wunder 2013, p. *w13746)*

This excerpt shows how medically assisted extension of fertility time is framed as something to be avoided, not only because of the neonatal and obstetrical risks but also because of possible disruption to intergenerational order. The flow of care descending the generations could be interrupted by a larger gap produced by the use of ‘social egg freezing’. This position is reluctant to change the continuity of family arrangements. Changing society seems desirable as long as it does not disrupt an intergenerational order facilitating women’s careers and concerns ‘only’ the place of women in society, allowing them to reconcile career and children more easily. Society should be transformed by social means, and reproductive medicine should not contribute to extending female fertility. In this sense, the use of RTs as fertility extension strategies is resisted, not because age-related fertility decline is a natural biological process and not a pathology (Dr. A.), but because these technologies are false remedies to societal transformations with undesirable medical and societal side-effects.

This last positioning shows another way of combining the biological, the individual and the social. Instead of rejecting the ‘social’ as an individual problem, such as in Dr. A.’s case, here the social is collective. It should be transformed in order for women to have more room to reconcile work and family. However, by resisting medically assisted extension of fertility, this position also reinforces the naturalness of the life course, of intergenerational order, and the gendered association of youth and fertility.

## Conclusions

By definition the ‘biological clock’ relates to a tension between the biological and social age norms of motherhood. Following Amir, the ‘biological clock’ set of discourses and practices can be analysed as a biotemporal regulatory mechanism for governing reproduction. It consists of ascribing to women’s biology – the fact of life of fertility decline – the social norms structuring reproductive lives temporally. These norms relate to the linearity of the life course, made up of a succession of crucial stages and limiting motherhood to a critical temporal window. They secure a heteronormative reproductive order by reinstating an injunction to motherhood and biologizing gender difference with regard to time, precisely in a historical moment when the order of gender and family relations is undergoing change. The biological clock discourse presumes that the biology of fertility decline is a fixed fact of life. However, when it gets technologized, its supposedly stable ground is disrupted, potentially extending the age limits of motherhood and contributing to a decoupling of biological and social age. Denaturalizing these limits leaves the door open for other kinds of normative logics to enter into play. What are the effects of this ‘culturalization’ of the biological? To what extent does it subvert or reinforce the temporal norms regulating gender and family formation?

Building on Amir’s feminist concept of biotemporality, this paper has presented a case study of the moral reasoning (Lakoff & Collier, 2004) of medical experts in Switzerland regarding the possibility of medically extending fertility. It argues that looking at their moral navigation is important for grasping how the ontological and the political are related. It shows that the possibility of using fertility extension technologies prompts them to question the role of medicine in societal transformations which lead women to have children later in life. The moral legitimacy and desirability of a medical intervention is discussed in regard to (a) the way they perceive the ontology of the fertility decline and thus of the age limits of motherhood, i.e. normal/pathological and natural/social; (b) a moral economy of responsibility which alternatively places the burden on the shoulders of women as individuals shoulders (position 1); on medicine to intervene (position 2); on society to change (position3) ; (c) the place of the social in reproductive biomedicine, whether biomedicine should adapt and adjust to societal transformations. While biological temporality might be troubled biotechnologically, the social temporal norms of the life course, both at the individual and intergenerational level, are brought back into the picture. These three ways of defining the ‘good’ of fertility extension shed light on the variability and complexity of medical experts’ reasoning, complicating a straightforward narrative of medicalisation as a homogeneous process. Biomedicalization is often criticized for biologizing and naturalizing social problems (Conrad, 1992). By seeking to understand the transformations brought about by the biomedicalization of reproductive ageing “from the inside out” (Clarke et al., 2003), the analysis insists on the variety of moral positionings which “culturalize” or “ethicize” the biological, and on the situated and relational work of disambiguating ontological binaries. Moreover, it sheds light on the paradoxical role of fertility extension in subverting and/or reinforcing gender and age norms especially in regard to both the heteronormative association between youth and fertility and the temporal normativity of the lifecourse and motherhood.

In the Swiss context, where access to RTs is possible only for medical reasons, the question about the fixed biology of age-limited motherhood brought about by the technological possibility of fertility extension, may lead to a strong distinction between what is normal and pathological being reaffirmed and to resistance to medical interventions for social reasons, tending thereby to biologise the social age limits of motherhood (Dr. A.). However, the distinction between the normal and the pathological becomes irrelevant when technological intervention to alleviate the suffering of childlessness is naturalized and medicine is envisioned as a help for women to adjust biological and social temporalities to each other, due to their lack of agency over the structural societal factors which cause them to have a child later in life (Dr. B and Dr. F). The legitimacy of medical intervention to extend fertility might also be resisted not in the name of a distinction between the normal and pathological, but due to the social causes of age-related infertility. According to this positioning, fertility extension RTs cause rather than solve medical and social problems. Reconfigurations in the intergenerational order should especially be avoided. In this way its naturalness and continuity are maintained, while patriarchal structures detrimental to women should be fought (Dr.W.).

This analysis supports Amir’s argument about the temporal regulation of reproduction through its biological dimension. However, it also shows how the naturalization logics shift when the biological is no longer taken for granted and is disrupted technologically, that is, when it becomes possible to decouple biological and social age. The characteristics of the biological as linear and irreversible, and of reproductive potential as a precious biological resource which needs to be carefully managed temporally, are at the core of the ‘biological clock’ discourse. What these three kinds of moral reasoning highlight is how a naturalistic understanding of time based on a specific understanding of the biological comes to the fore when biology is remade in ways which might trouble the regulatory temporal frame of gender relations, family formation and intergenerational social contract. However, depending on the moral reasoning configuration, they may strategically and alternatively be used to naturalize or denaturalize the age limits of motherhood and consequently legitimize or not the medical extension of fertility. The category of nature provides a powerful narrative to support both the intervention of ARTs for age-related infertility as a synchronizing or catching-up tool and to prevent it in the name of biological time. The analysis shows that to deepen our understanding of regulatory mechanisms at stake in the biotemporal governance of reproduction, it is important to look empirically at how experts understand the ontology of age-related infertility and relate it to a moral economy of responbility. When what is normal and natural for them is disrupted and shifts, it leaves the door open to think about the future of motherhood in which the potentiality of extending its time frame is carefully negotiated morally.

## Data Availability

Not applicable

## Footnotes

1 The notion of ‘after progress’ comes from Jain S.L and Kaufman S.R. 2011. Introduction to Special Issue. After Progress: Time and Improbable Futures in Clinic Spaces. *Medical Anthropology Quarterly* 25, 183–188.

2 It is important to note that I did not meet any doctor fully supporting the medically-assisted extension of female fertility. The doctors I met told me that the extension of fertility through ARTs was an object of contention among them, and my sample being limited, my analysis probably does not account for the whole variety of all existing positionings. All the doctors I met were in the French-speaking part of Switzerland. It is possible that it might be different in the German-speaking part. Dr. Zech, director of the Ovita clinics (http://www.ovita.eu/) would probably have another point of view, as would the intermediary agency Swixmed (http://www.swixmed.com/). However, I think that it was very difficult in Switzerland, at the time of my research, for medical practitioners to fully support PM pregnancies. As an example, I was told that Dr. Zech had been asked not to advertise his clinic in Switzerland. Further research on the moral reasoning of medical experts is needed.

3 All names of people met in the fieldwork are pseudonyms. Only when published papers are cited, are real names mentioned.

## References

[CR1] Adams V, Murphy M, Clarke AE (2009). Anticipation: Technoscience, life, affect, temporality. Subjectivity.

[CR2] Agamben, G. (1997). *Homo sacer. Le pouvoir souverain et la vie nue*. Seuil

[CR3] Amir M (2007). Bio-temporality and social regulation: The emergence of the biological clock. Polygraph: An International Journal of Culture and Politics.

[CR4] Antinori S, Versaci C, Gholami GH, Panci C, Caffa B (1993). Pregnancy: Oocyte donation in menopausal women. Human Reproduction.

[CR5] Baldwin K (2019). The biomedicalisation of reproductive ageing: Reproductive citizenship and the gendering of fertility risk. Health, Risk & Society.

[CR6] Baldwin, K. (2019b). *Egg freezing, fertility and reproductive choice: Negotiating responsibility, hope and modern motherhood*. Emerald Group Publishing

[CR7] Baldwin W, Nord C (1984). Delayed childbearing in the U.S.: Facts and fictions. Population Bulletin.

[CR8] Bamford, S. C., Bamford, S., & Leach, J. (2009). *Kinship and beyond: The genealogical model reconsidered*. Berghahn Books

[CR9] de Beauvoir, S. (1986). *Le deuxième sexe, tome 2*. Gallimard

[CR10] Billari, F. C. (2008). The postponement of childbearing in Europe: Driving forces and implications. *Vienna Yearbook of Population Research*, *2006*, 1–17. 10.1553/populationyearbook2006s1

[CR11] Bittner U, Eichinger T (2010). An ethical assessment of postmenopausal motherhood against the backdrop of successful antiaging medicine. Rejuvenation Research.

[CR12] Bleichenbacher M, Heitlinger E, Imthurn B (2010). La vision de la procréation médicalement assistée en Suisse. Bulletin Des Médecins Suisses| Schweizerische Ärztezeitung| Bollettino Dei Medici Svizzeri.

[CR13] Brown E, Patrick M (2018). Time, Anticipation, and the Life Course: Egg Freezing as Temporarily Disentangling Romance and Reproduction. American Sociological Review.

[CR14] Brown N (2003). Hope against hype—Accountability in biopasts, presents and futures. Science & Technology Studies.

[CR95] Bühler, N. (2021) *When reproduction meets ageing*. Emerald Publishing Limited.

[CR96] Bühler, N. (2022) The making of ‘old eggs’: The science of reproductive ageing between fertility and anti-ageing technologies. *Reproductive Biomedicine & Society Online*, 14169–181. 10.1016/j.rbms.2021.07.004 10.1016/j.rbms.2021.07.004 PMC8732751 35024473

[CR97] Bühler, N. (in press). When time becomes biological: The biomedicalisation of age-related infertility through the lens of women’s experiences of anticipation and ARTs. In V. Boydell and K. Dow (Eds.). *Technologies of reproduction across the lifecourse*. Emerald Press.

[CR94] Bühler, N., & Herbrand, C. (2022) Powering life through MitoTechnologies: Exploring the bio-objectification of mitochondria in reproduction. *BioSocieties*, 17(1) 99–122. 10.1057/s41292-020-00204-6

[CR15] Butler, J. (2006). *Gender trouble* (1st ed.). Routledge

[CR16] Campbell, P. (2011). Boundaries and risk: Media framing of assisted reproductive technologies and older mothers. *Social Science & Medicine (1982)*, *72*(2), 265–272. 10.1016/j.socscimed.2010.10.028 10.1016/j.socscimed.2010.10.028 21131118

[CR17] Canguilhem, G., & Foucault, M. (1991). *The normal and the pathological* (C. R. Fawcett, Trans.; First American Edition). Zone Books

[CR18] Cardona B (2008). ‘Healthy Ageing’ policies and anti-ageing ideologies and practices: On the exercise of responsibility. Medicine, Health Care, and Philosophy.

[CR19] Carroll K, Kroløkke C (2018). Freezing for love: Enacting ‘responsible’ reproductive citizenship through egg freezing. Culture, Health & Sexuality.

[CR20] Clarke, A. E., Mamo, L., Fosket, J. R., Fishman, J. R., & Shim, J. K. (Eds.). (2010). *Biomedicalization: Technoscience, health, and illness in the U*. S. Duke University Press

[CR21] Clarke AE, Shim JK, Mamo L, Fosket JR, Fishman JR (2003). Biomedicalization: Technoscientific Transformations of Health, Illness, and U.S. Biomedicine. American Sociological Review.

[CR22] Cohen J, Scott R, Alikani M, Schimmel T, Munné S, Levron J, Willadsen S (1998). Ooplasmic transfer in mature human oocytes. Molecular Human Reproduction.

[CR23] Collier, J. F., & Yanagisako, S. J. (1987). *Gender and kinship: Essays toward a unified analysis*. Stanford University Press

[CR24] Conrad P (1992). Medicalization and Social Control. Annual Review of Sociology.

[CR25] Engeli I (2009). The Challenges of Abortion and Assisted Reproductive Technologies Policies in Europe. Comparative European Politics.

[CR26] Law I, Dondorp W, de Wert G, Pennings G, Shenfield F, Diedrich K, ESHRE Task Force on Ethics and (2012). Oocyte cryopreservation for age-related fertility loss. Human Reproduction.

[CR27] Fassin, D. (2005). *1. L’ordre moral du monde essai d’anthropologie de l’intolérable*. p.17–50

[CR28] Fassin D (2006). La biopolitique n’est pas une politique de la vie. Sociologie et sociétés.

[CR29] Fassin, D. (2009). Moral economies revisited. *Annales. Histoire, Sciences Sociales*, *64th Year*(6), 1237–1266

[CR30] Fassin, D. (2012). *A companion to moral anthropology*. John Wiley & Sons

[CR31] FDA, C. (2018). for B. E. and. Advisory on legal restrictions on the use of mitochondrial replacement techniques to introduce donor mitochondria into reproductive cells intended for transfer into a human recipient. *FDA*. https://www.fda.gov/vaccines-blood-biologics/cellular-gene-therapy-products/advisory-legal-restrictions-use-mitochondrial-replacement-techniques-introduce-donor-mitochondria

[CR32] Flamigni C (1993). Egg donation to women over 40 years of age. Human Reproduction.

[CR33] Franklin, S. (1997). *Embodied progress: A cultural account of assisted conception*. Routledge

[CR34] Franklin, S. (1998). Making miracles: Scientific progress and the facts of life. In S. Franklin, & H. Ragoné (Eds.), *Reproducing reproduction. Kinship, power and technological innovation* (pp. 102–117). University of Pennsylvania Press

[CR35] Franklin, S. (2007). *Dolly mixtures: The remaking of genealogy*. Duke University Press

[CR36] Franklin, S. (2013). *Biological relatives: IVF, stem cells, and the future of kinship*. Duke University Press

[CR37] Franklin, S., & Lock, M. (Eds.). (2003). *Remaking life & death: Toward an anthropology of the biosciences*. School of American Research Press

[CR38] Friese, C. (2013). *Cloning wild life: Zoos, captivity, and the future of endangered animals*. NYU Press

[CR39] Friese C, Becker G, Nachtigall RD (2006). Rethinking the biological clock: Eleventh-hour moms, miracle moms and meanings of age-related infertility. Social Science & Medicine.

[CR40] Goold I, Savulescu J (2009). In Favour of Freezing Eggs for Non-Medical Reasons. Bioethics.

[CR41] Haraway D (1988). Situated Knowledges: The Science Question in Feminism and the Privilege of Partial Perspective. Feminist Studies.

[CR42] Harding, S. G. (2004). *The feminist standpoint theory reader: Intellectual and political controversies*. Psychology Press

[CR43] Jain SL (2007). Living in Prognosis: Toward an Elegiac Politics. Representations.

[CR44] Jamieson M (2016). The Politics of Immunity: Reading Cohen through Canguilhem and New Materialism. Body & Society.

[CR45] Johnson J, Canning J, Kaneko T, Pru JK, Tilly JL (2004). Germline stem cells and follicular renewal in the postnatal mammalian ovary. Nature.

[CR46] Katz S, Gish J (2015). Aging in the Biosocial Order: Repairing Time and Cosmetic Rejuvenation in a Medical Spa Clinic. The Sociological Quarterly.

[CR47] Katz S, Marshall BL (2004). Is the Functional ‘Normal’? Aging, Sexuality and the Bio-marking of Successful Living. History of the Human Sciences.

[CR48] Kaufman SR (2013). Fairness and the Tyranny of Potential in Kidney Transplantation. Current Anthropology.

[CR49] Kaufman SR, Fjord L (2011). Medicare, Ethics, and Reflexive Longevity: Governing Time and Treatment in an Aging Society. Medical Anthropology Quarterly.

[CR50] Kaufman SR, Morgan LM (2005). The Anthropology of the Beginnings and Ends of Life. Annual Review of Anthropology.

[CR51] Kortman, M., & Macklon, N. S. (2008). Oocyte donation in postmenopausal women: Medical and ethical considerations. *Obstetrics, Gynaecology & Reproductive Medicine*, 18(6), 168–169. 10.1016/j.ogrm.2008.04.005

[CR52] Kroløkke CH, Adrian SW (2013). Sperm on Ice. Australian Feminist Studies.

[CR53] Lakoff A, Collier SJ (2004). Ethics and the anthropology of modern reason. Anthropological Theory.

[CR54] Landecker H, Franklin S, Lock M (2003). On beginning and ending with apoptosis: Cell death and biomedicine. Remaking Life & Death: Toward an Anthropology of the Biosciences.

[CR55] Landecker, H. (2007). *Culturing life: How cells became technologies*. Harvard University Press

[CR56] Leridon H (2004). Can assisted reproduction technology compensate for the natural decline in fertility with age? A model assessment. Human Reproduction.

[CR57] Lock, M. M. (2001). *Twice dead*. University of California Press

[CR58] Lockwood, G. M. (2011). Social egg freezing: The prospect of reproductive ‘immortality’ or a dangerous delusion? *Symposium: Oocyte Cryopreservation*, *23*(3), 334–340. 10.1016/j.rbmo.2011.05.010 10.1016/j.rbmo.2011.05.010 21775211

[CR59] Manaï, D. (2008). La procreation médicalement assistée en droit Suisse: Vérité sur la conception et l’identité du donneur de gamètes. In B. Feuillet-Liger (Ed.), *Procréation médicalement assitée et anonymat: Panorama international* (pp. 261–273). Emile Bruylant

[CR60] Martin E (1991). The Egg and the Sperm: How Science Has Constructed a Romance Based on Stereotypical Male-Female Roles. Signs.

[CR61] Martin, E. (2001). *The woman in the body: A cultural analysis of reproduction* (Revised). Beacon Press

[CR62] Martin LJ (2010). Anticipating Infertility Egg Freezing, Genetic Preservation, and Risk. Gender & Society.

[CR98] Martin, L. J. (2017) Pushing for the perfect time: Social and biological fertility. *Women’s Studies International Forum*, 6291–98. 10.1016/j.wsif.2017.04.004

[CR63] Mertes H, Pennings G (2011). Social egg freezing: For better, not for worse. Reproductive Biomedicine Online.

[CR64] Moreira T, Palladino P (2008). Squaring the curve: The anatomo-politics of ageing, life and death. Body and Society.

[CR65] Morita Y, Tilly JL (1999). Oocyte Apoptosis: Like Sand through an Hourglass. Developmental Biology.

[CR66] Mykytyn CE (2008). Medicalizing the optimal: Anti-aging medicine and the quandary of intervention. Journal of Aging Studies.

[CR67] Oakley, A. (1972). *Sex, gender and society*. Maurice Temple Smith Ltd

[CR68] Pennings G (2002). Reproductive tourism as moral pluralism in motion. Journal of Medical Ethics.

[CR69] Pru JK, Tilly JL (2001). Programmed Cell Death in the Ovary: Insights and Future Prospects Using Genetic Technologies. Molecular Endocrinology.

[CR70] Rabinow P (1992). Studies in the Anthropology of Reason. Anthropology Today.

[CR71] Radin J (2013). Latent life: Concepts and practices of human tissue preservation in the International Biological Program. Social Studies of Science.

[CR72] Rose, N. (2007). *The politics of life itself: Biomedicine, power, and subjectivity in the twenty-first century*. Princeton University press

[CR73] Rose N (2009). Normality and Pathology in a Biomedical Age. The Sociological Review.

[CR74] Sauer MV, Paulson RJ, Lobo RA (1993). Pregnancy after age 50: Application of oocyte donation to women after natural menopause. The Lancet.

[CR75] Sauer, M. V., Paulson, R. J., & Lobo, R. A. (1995). Triplet pregnancy in a 51-year-old woman after oocyte donation. *American Journal of Obstetrics and Gynecology*, 172(3), 1044–1045. 10.1016/0002-9378(95)90043-8 10.1016/0002-9378(95)90043-8 7892847

[CR76] Smajdor A (2008). The ethics of egg donation in the over fifties. Menopause International.

[CR77] Smajdor, A. (2009). Between fecklessness and selfishness: Is there a biologically optimal time for motherhood?. In F. Simonstein (Ed.), *Reprogen-ethics and the future of gender* (43 vol., pp. 105–117). Springer. 10.1007/978-90-481-2475-6_9

[CR78] Sobotka T, Tremmel J (2010). Shifting Parenthood to Advanced Reproductive Ages: Trends, Causes and Consequences. A Young Generation Under Pressure?.

[CR79] Squier, S. M. (2004). *Liminal lives: Imagining the human at the frontiers of biomedicine*. Duke University Press

[CR80] Strathern, M. (1992a). *After nature: English kinship in the late twentieth century*. Cambridge University Press

[CR81] Strathern, M. (1992b). *Reproducing the future: Essays on anthropology, kinship and the new reproductive technologies*. Manchester University Press

[CR82] Taussig KS, Hoeyer K, Helmreich S (2013). The Anthropology of Potentiality in Biomedicine: An Introduction to Supplement 7. Current Anthropology.

[CR83] The Practice Committees of the American Society for Reproductive Medicine and the Society for Assisted Reproductive (2013). Mature oocyte cryopreservation: A guideline. Fertility and Sterility.

[CR84] Tilly J (1996). Apoptosis and ovarian function. Reviews of Reproduction.

[CR85] Tilly JL (2003). Oocyte apoptosis: Prevention strategies, and implications for female aging and the menopause. Annales D’endocrinologie.

[CR86] Van De Kaa DJ (1987). Europe’s second demographic transition. Population Bulletin.

[CR87] van de Wiel L (2015). Frozen in anticipation: Eggs for later. Women’s Studies International Forum.

[CR91] van de Wiel, L. (2020). *Freezing fertility: Oocyte cryopreservation and the gender politics of aging*. NYU Press 33661591

[CR88] Wainwright, S. P., Williams, C., Michael, M., Farsides, B., & Cribb, A. (2007). Ethical boundary-work in the embryonic stem cell laboratoy. In R. de Vries, L. Turner, K. Orfali, & C. L. Bosk (Eds.), *The view from here. Bioethics and the social sciences* (pp. 67–82). Blackwell

[CR89] Waldby, C. (2014). ‘Banking time’: Egg freezing and the negotiation of future fertility. *Culture, Health & Sexuality*, 1–13. 10.1080/13691058.2014.951881 10.1080/13691058.2014.951881 25247927

[CR90] Warin M, Zivkovic T, Moore V, Davies M (2012). Mothers as smoking guns: Fetal overnutrition and the reproduction of obesity. Feminism & Psychology.

[CR92] Woods DC, Tilly JL (2015). Autologous Germline Mitochondrial Energy Transfer (AUGMENT) in Human Assisted Reproduction. Seminars in Reproductive Medicine.

[CR93] Wunder D (2013). Social freezing in Switzerland and worldwide—A blessing for women today?. Swiss Medical Weekly.

